# Versatile chemical handling to confine radioactive cesium as stable inorganic crystal

**DOI:** 10.1038/s41598-018-32943-9

**Published:** 2018-10-09

**Authors:** Nguyen Duy Quang, Hiromi Eba, Kenji Sakurai

**Affiliations:** 1 National Institute for Material Science, 1-2-1, Sengen, Tsukuba, Ibaraki, 305-0047 Japan; 2 Vietnam Atomic Energy Institute, Nuclear Research Institute, Da Lat, Vietnam; 3 Department of Chemistry and Energy Engineering, Graduate School of Engineering, Tokyo City University, 1-28-1 Tamazutsumi, Setagaya, Tokyo 158-8557 Japan

## Abstract

The present paper describes an extremely efficient, reproducible and inexpensive chemical handling method for converting the nuclear wastes contaminated by radioactive cesium to stable inorganic crystal, pollucite (CsAlSi_2_O_6_), which is promising as a form of the final storage. In this processing, the clays are used as a source for aluminum and silicon, and it is important to get a well-mixed homogenous solution by the aid of some heat and pressure. The present method proposes the use of ethylene glycol as a solvent, rather than water. It has been found that one can obtain crystalline pollucite by heating up to 350 °C in a high-pressure container (~15 MPa), mixed with montmorillonite – an abundant natural clay and ethylene glycol. It has been found that the reduction of the amount of water helps to achieve very high confinement rate in a reasonable time of few~20 h. This will be fairly important in processing contaminated water in the nuclear power plant. The influence of seawater has been also examined.

## Introduction

Radioactive ^137^Cs is readily produced in large quantities as a fission product in a nuclear power plant based on nuclear fission of ^235^U. If it is released into the environment, mainly as a result of nuclear accidents, it will cause significant concerns in terms of the public health risks. As the lifetime of ^137^Cs is quite long (about 30.17 years), the contaminated area will remain dangerous for residence purposes for many years when the dose rate of the radiation is extremely high^1^. Therefore, particularly after critical accidents in the recent past^2–5^, a variety of research has been reported mainly on technologies for decontaminating radioactive waste from the environment, i.e., soil in fields, sediment in ponds, as well as contaminated water. Many efficient methods suitable for absorption/ adsorption of ^137^Cs have been proposed^6–8^, and the mechanisms have been also studied by magic angle spinning nuclear magnetic resonance (MAS NMR), X-ray absorption fine structures (XAFS) and other methods^9–13^. On the other hand, it is extremely important to establish the method to immobilize and store ^137^Cs in a stable form, because this alkaline metal is quite easily dissolved in water and can spread again^14–16^.

The present paper reports an extremely efficient and simple method to confine the ^137^Cs as a stable inorganic crystal. As described later, it has been found that one can obtain crystalline pollucite (CsAlSi_2_O_6_)^17,18^ by heating up to 350 °C in a high-pressure container (6–17 MPa), mixed with a natural abundant clay material and inexpensive solvent, which are montmorillonite (MMLT, (Na, Ca)_0.33_(Al, Mg)_2_ Si_4_O_10_(OH)_2_-nH_2_O) and ethylene-glycol (EG), respectively. As the present research is a kind of feasibility study, pure CsCl chemical containing non-radioactive Cs is used as a source of Cs, instead of real nuclear wastes.

## Results

### Solvothermal treatment to immobilize Cs

**Figure 1:**
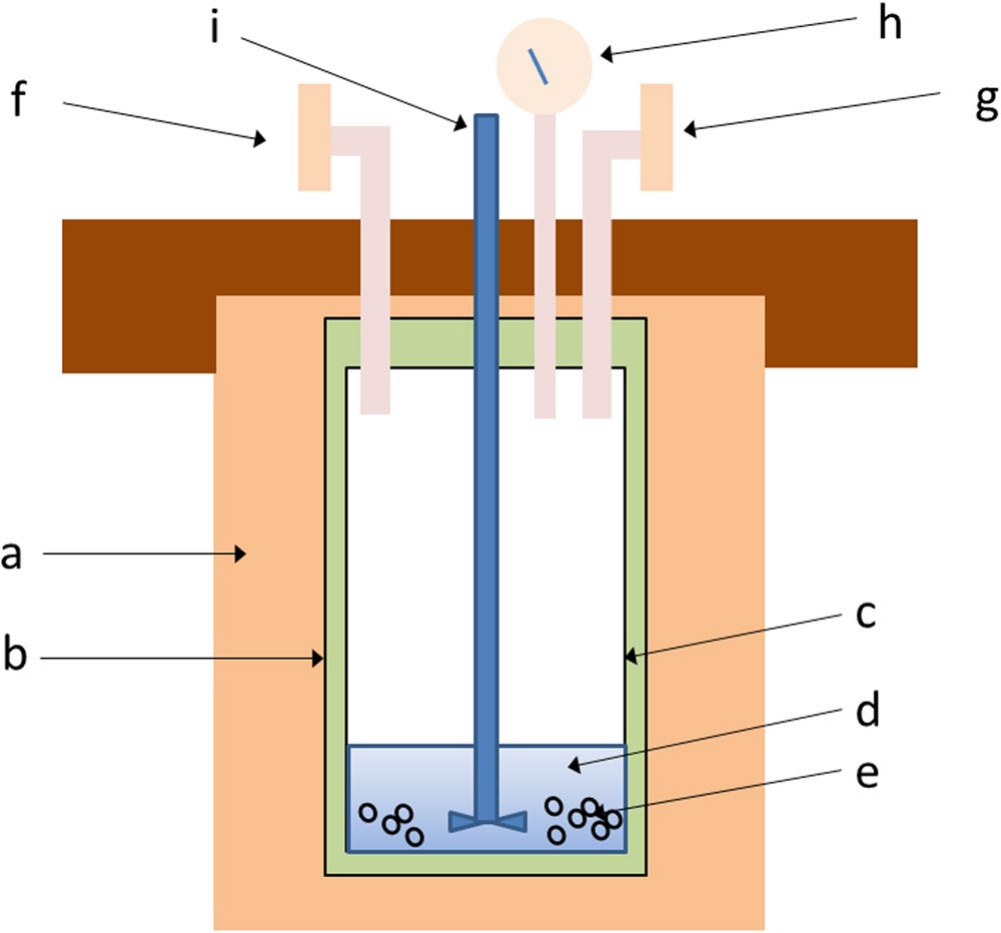
Schematics of the prototype reactor for the solvothermal treatment of radioactive waste to immobilize Cs as a stable crystalline pollucite. (**a**) Heater, (**b**) Container (outer part, capacity volume of 200 ml), (**c**) Container (inner part), (d) Solvent (EG) and liquid waste (such as sea water including Cs) with small addition of NaOH (typical total volume is 30 ml), (**e**) Mixture of MMLT (typical amount is 0.25 g) and solid waste containing Cs, (**f**,**g**) Gas ports, (**h**) Pressure gauge, (**i**) Stirrer.

Figure 1 schematically shows a prototype reactor, which can process the radioactive waste containing ^137^Cs. Before starting to use this reaction, some pre-concentrating procedure is required. When the waste is solid, crushing and pulverizing it to a fine powder is desirable. If the waste is liquid, such as water, reducing the volume by evaporation is recommended. The method is so-called solvothermal treatment^19–22^, which uses both a high-pressure and heating device to synthesize the inorganic crystalline materials at relatively low temperatures such as 300–400 °C. To maintain high pressure up to 20 MPa under such temperature condition, some metallic seals are employed. This treatment is a kind of mixture of two well-known and established synthesis techniques, the hydrothermal and the sol-gel methods. As is often the case for quite a long reaction time at rather high temperature and pressure, some black polymeric matter is obtained in the present study. This appears very similar to some dispersed nano-particles, which are also prepared by solvothermal treatment, to be used as ink for printing^23,24^. Although the solvothermal treatment has such capability, such ink-like material was not used for the present processing of Cs. The reactor shown in Fig. 1 appears similar to that of hydrothermal method, but the main difference is that the solution is not aqueous, leading to development of new chemical reaction which have been regarded as almost impossible^22^. In 2014 Yokomori *et al*. reported their pioneering research on hydrothermal storage of Cs as pollucite^15^, and other research group also studied the feasibility^16^. Whilst examining their work, it has been that clay, which is used as a source of aluminum and silicon, is much more easily dissolved in ethylene glycol than water, under similar temperature and pressure conditions. As is shown later, the solvothermal treatment allows fairly stable and reproducible processing, and can give better results by reducing the amount of water. During a series of experiments, it has been found that the crystalline pollucite is not obtained when water is used instead of ethylene-glycol, as long as all other conditions are maintained as described in the Methods section. The reason is just that montmorillonite is not easily dissolved into water. Therefore the solvothermal treatment is much more suitable than the hydrothermal procedure for the same goal to confine Cs collected from nuclear waste in the stable inorganic crystal such as pollucite. In the present research, it has been found that reduction of water amount in the sample is crucial to get a single phase pollucite reproducibly, though a few percent water to ethylene-glycol is still acceptable. In practice, controlling the amount of the water contained in the nuclear wastes is necessary before the solvothermal treatment.

### Identification of the material obtained by solvothermal treatment

**Figure 2:**
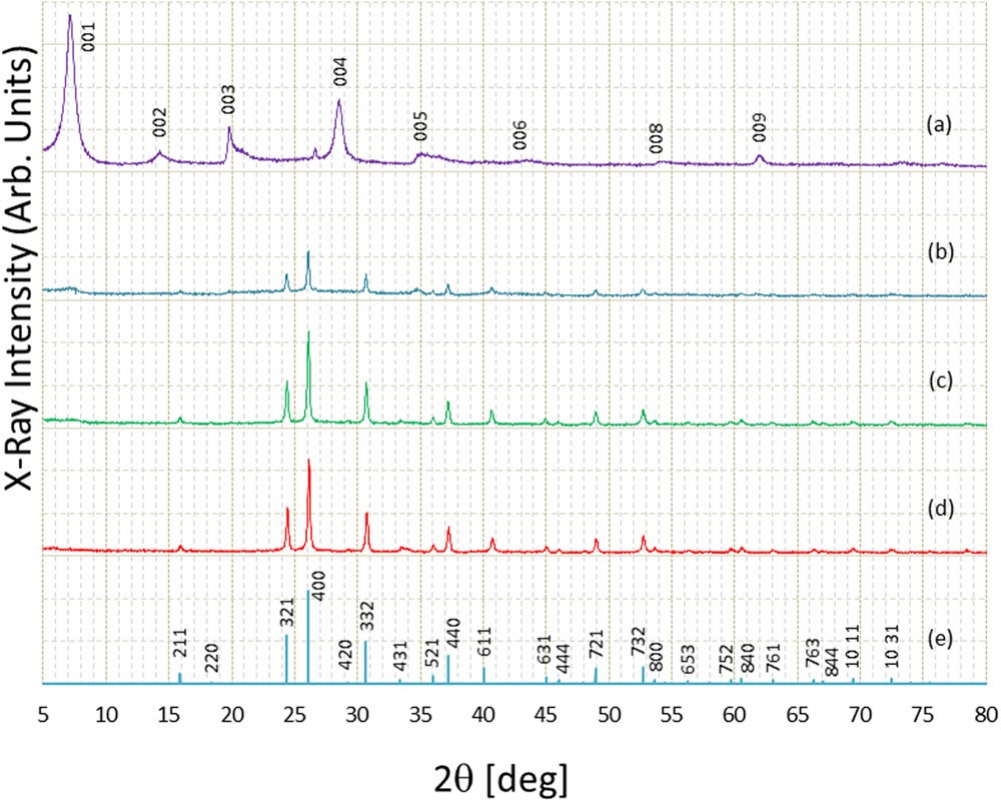
Changes of X-ray diffraction pattern by solvothermal treatment. (**a**) MMLT (before treatment), (**b**) 320 °C, 6.0 MPa, (**c**) 320 °C 8.7 MPa, (**d**) 350 °C, 14.8 MPa and (**e**) data taken from the database (ICDD PDF card 01-088-0055)^17^.

After closing all valves of the reactor (shown in Fig. 1), the sample (a mixture of CsCl, MMLT and EG, with small amount of NaOH, see Method section for more details) was heated to 350 °C, causing an increase in pressure, from 1.8–14.8 MPa. The heating time was 3.0–20.6 h. After the reaction completed, the product was collected and cleaned by ethanol. The finally obtained solid powder was dried in air at room temperature for about 30 min. Figure 2 shows some typical X-ray diffraction (XRD) patterns. In the beginning, the solid material in the container is only MMLT. As shown in (a) in Fig. 2, one can see quite strong peaks in the low angle part (7.10 deg for 001) and a series of peaks at 14.26 deg, 19.8 deg, 28.56 deg, 35.12 deg etc for 002, 003, 004 and 005 reflections, respectively. Such XRD patterns are likely due to clay material, and the peaks correspond to stacking structure. The inter-layer distance has been estimated as 1.23 ± 0.02 nm. During the solvothermal treatment, MMLT is dissolved into EG because of high temperature and high pressure, and such layer structures are completely destroyed, leading to the release of Al^3+^ and Si^4+^ into the solvent. The solution contains all necessary elements, Al, Si, and Cs to synthesize a pollucite crystal. As shown in (b) in Fig. 2, in the sample processed at 320 °C and 6.0 MPa, the series of MMLT peaks became faint, while many new peaks appeared. In (c) and (d), the new peaks grew further, and no remaining MMLT was found. As clearly seen, the XRD patterns for the samples processed at 320 °C and 8.7 MPa (c) and at 350 °C and 14.8 MPa match well with that of the cubic phase of pollucite crystal (space group *Ia-3d*, a = 1.3665 nm)^17^. On the other hand, at 300 °C, the maximum pressure remaining was only around 2 MPa, which was not sufficient for the reaction.

### Confinement of Cs in the obtained crystal

**Figure 3:**
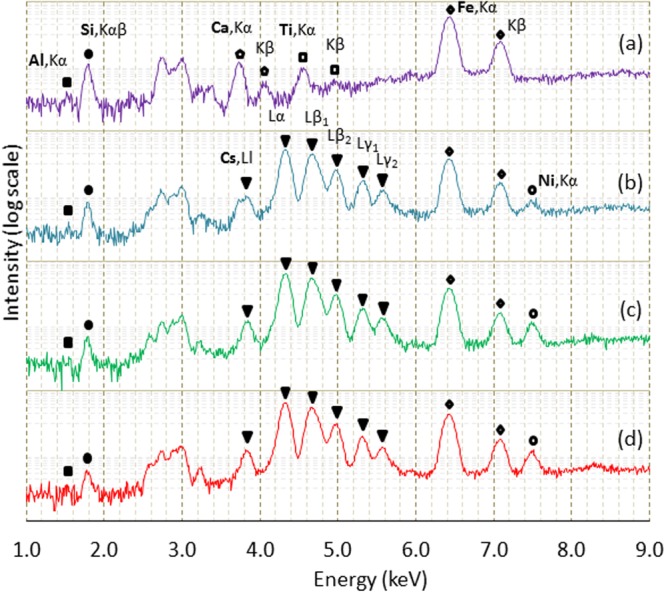
Changes of X-ray fluorescence spectra by solvothermal treatment. Al (closed square), Si (closed circle), Cs (closed triangle), Ca (open pentagon), Ti (open square), Fe (open diamond) and Ni (open oval). (**a**) MMLT (before treatment), (**b**) 320 °C, 6.0 MPa, (**c**) 320 °C 8.7 MPa, and (**d**) 350 °C, 14.8 MPa.

It is also important to see the chemical composition of the obtained powder. Figure 3 shows some typical X-ray fluorescence (XRF) spectra taken in air. The detailed chemical composition of the MMLT used in the present experiment is described elsewhere^25^. When the chemical composition is expressed as oxide form, it contains SiO_2_ of 54.0 wt%, Al_2_O_3_ of 19.9 wt%, MgO of 3.0 wt%, Fe_2_O_3_ of 1.9 wt%, CaO of 0.4 wt%, K_2_O of 0.4 wt%, and TiO_2_ of 0.1 wt%. As the MMLT is a natural product, it contains many impurities, such as Ti and Fe. As shown in Fig. 3(a), one can confirm the existence of Al, Si, Ca, Ti and Fe. It may be possible to detect K, but it is fairly weak mainly because of attenuation in the air path. As Mg K lines are in such a low energy region, detection is almost impossible. Compared with MMLT, first of all, the finally obtained powders (shown in (b), (c) and (d)) clearly contain Cs. This indicates that Cs is no doubt confined in the obtained crystalline powder. As Al and Si are also seen, the data provides further solid support to confirm that the obtained material is pollucite. One cannot see Ca and Ti peaks, which are contained in the MMLT. Such elements remained in the solution. On the other hand, Fe and Ni were found in the spectra. A small amount of Fe may occupy the Al site in the pollucite crystal. On the other hand, the source of Ni would be the container of the autoclave made of stainless steel. This could indicate that the final material obtained through the present solvothermal treatment has some impurities.

### Influence of addition of seawater

**Figure 4:**
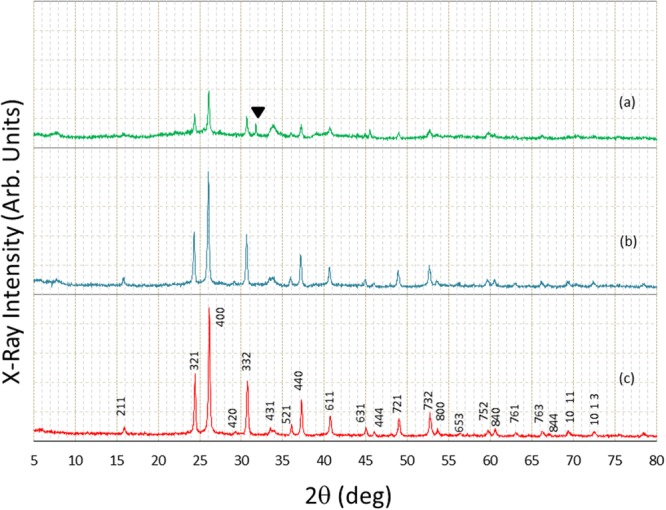
Comparison of X-ray diffraction patterns among samples synthesized with and without artificial seawater. The temperature was set as 350 °C. (**a**) addition of artificial seawater with 3 times the concentration of natural seawater, (**b**) addition of artificial seawater of the same concentration as natural one, (**c**) without addition of artificial seawater. The triangle mark represents unknown new peak, which is not from crystalline pollucite.

To assess feasibility when processing real nuclear waste, addition of artificial seawater has been attempted. The addition of the artificial sea water is set at less than 10% of the volume of EG, in order to treat the samples with similar temperature and pressure conditions. The present solvothermal treatment assumes some prior concentrating procedure for the waste, before considering final storage. As a result, one of the most important questions is the influence of major elements in seawater, which will be a part of the concentrated nuclear waste. Figure 4 shows the influence of addition of artificial seawater. As clearly seen in Fig. 4(a), it is still possible to get pollucite even with the addition of artificial seawater with 3 times the concentration of natural seawater. Though main feature of the X-ray diffraction patter is still close to that of pollucite crystal, for example, some new peak appears at around 31.74°. The confinement of Cs is not perfect, and some Cs is still remained in the solution. Because of the existence of seawater, the maximum pressure became higher than usual (16.2 MPa and 16.9 MPa for Fig. 4(a,b), respectively), leading to some difficulties in obtaining pollucite crystal efficiently and stably. When large amounts of nuclear waste in the form of aqueous solution are provided for processing, first, it is important to reduce the amount by evaporation. Another important point is the concentration of Cs in the waste. After quantitative analysis, the amount of MMLT used for the reaction needs to be determined so that the mole ratio of Cs:Al:Si = 1:1:2.

## Discussions

During the present research, the following points have been found: cesium can be confined as a stable inorganic crystal, pollucite (CsAlSi_2_O_6_), by the solvothermal treatment using MMLT and EG; (i) when heated to 350 °C, the maximum pressure reaches 14.8 MPa, and pollucite is easily formed within approximately 10 h, (ii) when heating is stopped at 320 °C, the pressure reaches 6.0 MPa and 8.7 MPa after 9.6 h and 20.6 h, respectively, and the latter condition is preferable, (iii) when heating is stopped at 300 °C, the maximum pressure remains as low as around 2 MPa, and synthesis is not very successful. In the pioneering work^15^ by Yokomori *et al*., the method is a hydrothermal procedure, and uses water, instead of EG. As different sources (such as bentonite) for Al and Si are used, they also needed other additives such as AlCl_3_. Compared with the hydrothermal procedure, the present method is much more stable and reproducible. In reality, the processing of contaminated water in a nuclear power plant could be demanded. It is highly recommended to reduce the volume (the ratio to the EG solvent), and to enrich the concentration of Cs by chemical methods. Then the present technique will help to process such highly-concentrated nuclear waste by confining radioactive Cs in the stable crystal. The feasibility of the scheme has been well examined in the laboratory.

In summary, an extremely efficient and simple method to store radioactive Cs contained in nuclear waste as a stable inorganic crystal has been demonstrated. A prototype reactor has been developed. The method employed is solvothermal treatment using ethylene glycol as a solvent. The method uses montmorillonite as a source of Si and Al to synthesize the pollucite crystal (CsAlSi_2_O_6_). It is important to adjust the mole ratio as Cs:Al:Si = 1:1:2 in the source material. The pollucite crystal is reproducibly obtained at 320~350 °C, and at 8.7~14.8 MPa. The typical reaction time is around 10~20.6 h. The influence of the addition of artificial seawater is also examined. The present method still works, if the volume ratio to the EG solvent is within 10% and the concentration is as much as 3 times that of natural seawater.

The authors gratefully acknowledge the valuable advice of Dr. Hirohisa Yamada (National Institute for Material Science) on the use of a standard montmorillonite sample. The authors’ thanks also go to Ms. Yen Pham, and Ms. Megumi Iwamoto for their cooperation and assistance in the early stage of the present research. One of the authors (N.D.Q.) wishes to thank the Nuclear Researcher Exchange Program of the Japanese government for financial support. The work was done during his leave from the Vietnam Atomic Energy Institute, Nuclear Research Institute, Da Lat, Vietnam.

## Methods

### Chemicals

As the present work is a kind of feasibility study for the proposed scheme, pure CsCl powder (Wako Chemical, purity 99.0%) containing only non-radioactive Cs was used for the experiment. To make pollucite crystal, one would need a source for Al and Si. In the present research, MMLT produced in the vicinity of Mt. Tsukinuno, Yamagata, Japan (available as the standard sample JCSS3101 from the Japan Clay Science Society)^25^ was employed, as the mole ratio of Al and Si is close to 1:2. MMLT (0.25 g), CsCl (0.25 g) and EG (Wako Chemical, purity 99%, density 1.112~1.118 g/ml at 20 °C) of typically 30 ml were mixed and put into the container in the reactor. In the present procedure, controlling the chemical composition of the starting material is extremely important, because the mole ratio of Cs, Al, and Si is ideally 1:1:2 in crystalline pollucite, which is a final product of this reaction. While CsCl is easily dissolved in EG even before starting any reaction, MMLT remained as a powder. It has been found that addition of NaOH granular (Wako Chemical, purity 97%) of 0.02 g effectively stabilizes the synthesis. To see the reproducibility of the optimized condition, the color change of commercial pH paper (WHATSMAN-BDH Full Range pH 1–14) was monitored. Addition of 0.02 g NaOH leads to turning the color to light grass green (corresponding to pH of 8~9 in the case of aqueous solution). To study the feasibility of the present procedure in the sea water case, addition of artificial sea water (Daigo’s Artificial Seawater SP for Marine Microalgae Medium, Nihon Pharmaceutical Co. Ltd.) was attempted.

The detailed chemical composition of artificial seawater is tabulated in Table 1, indicating that NaCl, MgCl_2_.6H_2_O, Na_2_SO_4_ and CaCl_2_.2H_2_O are major components. As the artificial sea water is provided as powder (of 36 g), 1000 ml of pure water was used for the experiment. In addition, 3 times concentrated seawater was prepared by 300 ml of pure water for the same amount of the powder.

**Table 1:** Chemical composition of artificial seawater powder (36 g for 1 L).

Composition	content (mg)
NaCl	20,747
MgCl_2_.6H_2_O	9,474
CaCl_2_.2H_2_O	1,326
Na_2_SO4	3,505
KCl	597
NaHCO_3_	171
KBr	85
Na_2_B_4_O_7_.10H_2_O	34
SrCl_2_	12
NaF	3
LiCl	1
KI	0.07
CoCl_2_.6H_2_O	0.0002
AlCl_3_.6H_2_O	0.008
FeCl_3_.6H_2_O	0.005
Na_2_WO_4_.2H_2_O	0.0002
(NH_4_)_6_Mo_7_O_24_.4H_2_O	0.02
MnCl_2_.4H_2_O	0.0008
Total	35,955

### Characterization

To identify the crystal structure and the chemical composition of the finally obtained product, X-ray diffraction (Rigaku Ultima-III, Cu Kα, 40 kV - 30 mA) and X-ray fluorescence (Oxford Series 5000 Rh tube, 30 kV - 0.5 mA, Amptek 123-SDD detector) analyses have been achieved.
